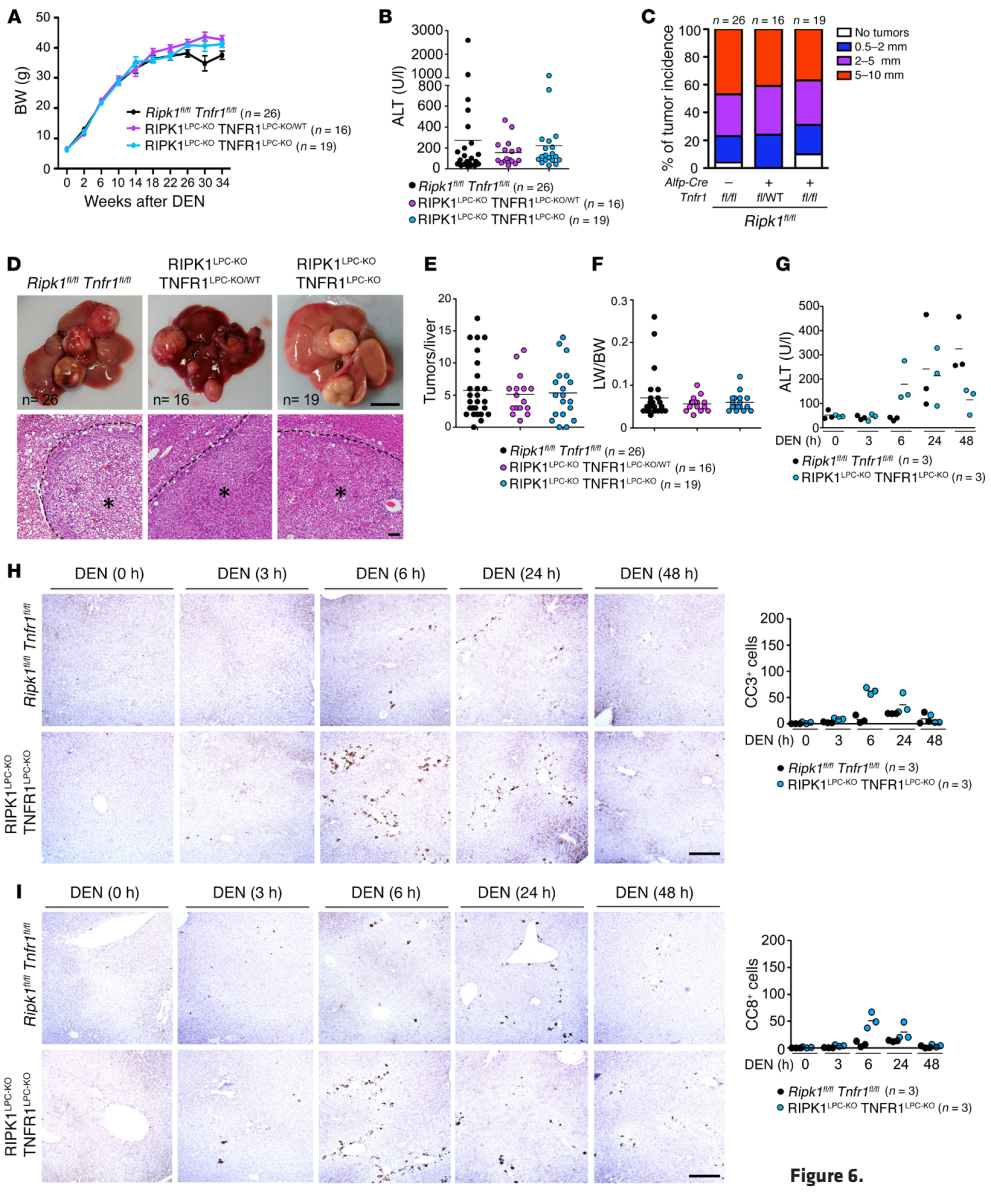# Corrigendum to Kinase-independent functions of RIPK1 regulate hepatocyte survival and liver carcinogenesis

**DOI:** 10.1172/JCI202164

**Published:** 2025-12-01

**Authors:** Trieu-My Van, Apostolos Polykratis, Beate Katharina Straub, Vangelis Kondylis, Nikoletta Papadopoulou, Manolis Pasparakis

Original citation: *J Clin Invest*. 2017;127(7):2662–2677. https://doi.org/10.1172/JCI92508

Citation for this corrigendum: *J Clin Invest*. 2025;135(23):e202164. https://doi.org/10.1172/JCI202164

The authors recently became aware of the following errors in the original manuscript: Figure 6D contained images duplicated from 7E; in Figure 6H, the DEN (0 h), RIPK1^LPC-KO^ TNFR1^LPC-KO^ image was derived from a DEN (48 h), RIPK1^LPC-KO^ TNFR1^LPC-KO^ sample; and in Figure 6I, the DEN (0 h) RIPK1^LPC-KO^ TNFR1^LPC-KO^ image was from a DEN (3 h) *Ripk1^fl/fl^* *Tnfr1^fl/fl^* sample. The corrected figure, based on the original source data, is provided below. The HTML and PDF versions of the paper have been updated. The authors have stated that the errors in representative image selection did not affect the quantifications presented.

The authors regret the errors.

## Figures and Tables

**Figure 6:**